# 2097: Aging-associated increases in platelet granzyme A regulate pro-inflammatory gene synthesis by monocytes

**DOI:** 10.1017/cts.2017.201

**Published:** 2018-05-10

**Authors:** Matthew Thomas Rondina, Robert A. Campbell, Anish Bhatnagar, Zechariah Franks, Jesse W. Rowley, Bhanu Kanth Manne, Mark A. Supiano, Alistair N. Ward

**Affiliations:** The University of Utah School of Medicine, Salt Lake City, UT, USA

## Abstract

OBJECTIVES/SPECIFIC AIMS: Platelets govern signal-dependent inflammatory responses by leukocytes. Although dysregulated inflammation is common in older adults, platelet-leukocyte signaling events and downstream inflammatory gene synthesis in aging is not known. METHODS/STUDY POPULATION: Highly-purified platelets and monocytes were isolated from healthy older (age>60, n=27) and younger (age<45, n=36) adults and incubated together in autologous and nonautologous conditions. Inflammatory gene synthesis by monocytes, basally and in the presence of activated platelets, was examined. Next-generation RNA-sequencing allowed for unbiased profiling of the platelet transcriptome in older and younger adults. Differentially expressed candidates in aged platelets were validated and recombinant granzyme A (in the presence and absence of TLR4 and Caspase-1 inhibition) identified putative ligands controlling inflammatory gene synthesis. RESULTS/ANTICIPATED RESULTS: In unstimulated or activated conditions, monocyte chemoattractant protein 1 (MCP-1) and interleukin-8 (IL-8) synthesis by monocytes alone did not differ between older and younger adults. However, in the presence of autologous activated platelets, monocytes from older adults synthesized significantly greater MCP-1 (867.150 vs. 216.36 ng/mL, *p*<0.0001) and IL-8 (41.5 vs. 9.2 ng/mL, *p*<0.0001) than younger adults. Nonautologous, or switch experiments, demonstrated that aged platelets were sufficient for upregulating MCP-1 and IL-8 synthesis by monocytes. Surprisingly, classic platelet proteins known to signal to monocytes and induce MCP-1 synthesis (p-selectin, RANTES, and PF4) were not increased in platelets from older adults. Using RNA-seq followed by validation via RT-PCR and immunoblot, we identified candidate platelet molecules increased in aging that mediate platelet-monocyte signaling and pro-inflammatory gene synthesis. We confirmed that granzyme A (GrmA), a serine protease not previously identified in platelets, is present in human platelets at the mRNA and protein level. GrmA is secreted by activated platelets in signal-dependent fashion. Moreover, GrmA in platelets is significantly increased in aging (~9-fold vs. younger adults). Blocking GrmA inhibited MCP-1 and IL-8 synthesis in older adults. Finally, we uncovered that platelet GrmA signaling to monocytes is regulated through TLR4 and Caspase-1. DISCUSSION/SIGNIFICANCE OF IMPACT: Human aging is associated with reprogramming of the platelet transcriptome. A previously unrecognized protein in platelets, GrmA, is increased in aging and causes increased MCP-1 and IL-8 gene synthesis by target monocytes in a TLR4 and Caspase-1 dependent mechanism. Increased platelet GrmA in aging may contribute to injurious inflammatory responses common in older adults.

